# Targeting cMET with INC280 impairs tumour growth and improves efficacy of gemcitabine in a pancreatic cancer model

**DOI:** 10.1186/s12885-015-1064-9

**Published:** 2015-02-19

**Authors:** Franziska Brandes, Katharina Schmidt, Christine Wagner, Julia Redekopf, Hans Jürgen Schlitt, Edward Kenneth Geissler, Sven Arke Lang

**Affiliations:** Department of Surgery, University Hospital Regensburg, University of Regensburg, Franz-Josef-Strauss Allee 11, 93053 Regensburg, Germany

**Keywords:** Pancreatic cancer, cMET, Resistance, Survival

## Abstract

**Background:**

Expression and activation of the cMET receptor have been implicated in tumor progression and resistance to chemotherapy in human pancreatic cancer. In this regard we assessed the effects of targeting cMET in pancreatic cancer models *in vitro* and *in vivo*.

**Methods:**

Human (L3.6pl, BxP3, HPAF-II, MiaPaCa2) and murine (Panc02) pancreatic cancer cell lines, endothelial cells (ECs) and vascular smooth muscle cells (VSMCs) were used for the experiments. Furthermore, the human pancreatic cancer cell line MiaPaCa2 with acquired resistance to gemcitabine was employed (MiaPaCa2(G250)). For targeting the cMET receptor, the oral available, ATP-competitive inhibitor INC280 was used. Effects of cMET inhibition on cancer and stromal cells were determined by growth assays, western blotting, motility assays and ELISA. Moreover, orthotopic xenogeneic and syngeneic mouse (BALB-C nu/nu; C57BL/6) models were used to assess *in vivo* efficacy of targeting cMET alone and in combination with gemcitabine.

**Results:**

Treatment with INC280 impairs activation of signaling intermediates in pancreatic cancer cells and ECs, particularly when cells were stimulated with hepatocyte growth factor (HGF). Moreover, motility of cancer cells and ECs in response to HGF was reduced upon treatment with INC280. Only minor effects on VSMCs were detected. Interestingly, MiaPaCa2(G250) showed an increase in cMET expression and cMET inhibition abrogated HGF-induced effects on growth, motility and signaling as well as DFX-hypoxia HIF-1alpha and MDR-1 expression *in vitro. In vivo*, therapy with INC280 alone led to inhibition of orthotopic tumor growth in xenogeneic and syngeneic models. Similar to *in vitro* results, cMET expression was increased upon treatment with gemcitabine, and combination of the cMET inhibitor with gemcitabine improved anti-neoplastic capacity in an orthotopic syngeneic model. Immunohistochemical analysis revealed a significant inhibition of tumor cell proliferation (Ki67) and tumor vascularization (CD31). Finally, combination of gemcitabine with INC280 significantly prolonged survival in the orthotopic syngeneic tumor model even when treatment with the cMET inhibitor was initiated at an advanced stage of disease.

**Conclusions:**

These data provide evidence that targeting cMET in combination with gemcitabine may be effective in human pancreatic cancer and warrants further clinical evaluation.

**Electronic supplementary material:**

The online version of this article (doi:10.1186/s12885-015-1064-9) contains supplementary material, which is available to authorized users.

## Background

Pancreatic cancer is the fourth leading cause for cancer-related death in Europe and the U.S and therefore represents one of the most aggressive malignancies [1,2]. Since pancreatic cancer is often diagnosed in locally advanced or metastatic stages, surgery as the only curative option is possible in only 10–20% of patients [3]. So far, gemcitabine is considered the standard chemotherapy for advanced pancreatic cancer [3]. Although the combination of 5-fluorouracil, leucovorin, irinotecan and oxaliplatin (FOLFIRINOX) recently showed an extension of life by 4 months when compared to gemcitabine, this regime has severe side effects and, therefore, is only applicable for very few patients [4]. In consequence new therapeutic options based on the molecular biology of pancreatic cancer are urgently needed to improve the survival of patients.

The receptor tyrosine kinase cMET and its ligand HGF (hepatocyte growth factor) play an important role in embryogenesis and tissue regeneration [5-7]. Binding of HGF to its corresponding receptor cMET leads to activation of intracellular signalling pathways including MAPK/ERK, PI3K/AKT and FAK (reviewed in [8]). In cancer, this confers multiple effects such as resistance to chemotherapy, induction of angiogenesis and promotion of metastasis (reviewed in [9]). With regards to pancreatic cancer, expression of cMET has been associated with poor survival [10] and phosphorylation of cMET has been described in patients with early distant metastases even after complete surgical resection [11]. Moreover, involvement of cMET activation in resistance to gemcitabine therapy [12], tumour cell motility [13] and secretion of angiogenic factors [14] has been reported in pancreatic cancer. Therefore, targeting cMET might be a promising approach for anti-neoplastic therapy in this devastating tumour entity.

INC280 [(also known as INCB028060); 2-fluoro-N-methyl-4-(7-(quinolin-6-ylmethyl)imidazo[1,2-b][1,2,4]triazin-2-yl)benzamide] is an orally available, small molecule ATP competitive inhibitor of cMET. It is selective for cMET, but also impairs positive (cMET-mediated) regulation of EGFR (epidermal-growth factor receptor) and has shown potent anti-neoplastic activity in preclinical studies [15]. In addition, a dose-escalating clinical phase I study showed manageable toxicity and promising dose-dependent decreases in cMET phosphorylation (NCT01072266). Currently, phase I and II studies for patients with advanced solid malignancies (NCT01911507, NCT01546428, NCT01324479), hepatocellular carcinoma (HCC), non-small cell lung cancer (NSCLC), renal cell carcinoma and melanoma have been launched (NCT01737827, NCT01610336, NCT01820364). Hence, targeting cMET with INC280 might also be a promising treatment option for human pancreatic cancer.

In the present study, we assessed the anti-neoplastic activity of targeting cMET with INC280 in pancreatic cancer models and found substantial *in vitro* inhibition of HGF-induced cancer cell motility and –signaling, as well as reversal of resistance-mediating properties. To validate our findings *in vivo*, we initially used an orthotopic xenogeneic mouse model and, subsequently, an orthotopic syngeneic mouse model, since the latter model harbors a functional immune system. Evaluation of INC280 in combination with gemcitabine on tumour growth in these experimental murine models provide *in vivo* evidence that targeting cMET has potential that could be applied to improve outcomes in patients with pancreatic cancer.

## Methods

### Cell culture and reagents

Human pancreatic cancer cell lines BxPC-3, MiaPaCa2, HPAF-II (American Type Culture Collection), L3.6pl (kindly provided by Dr. I. J. Fidler (The University of Texas M.D. Anderson Cancer Center)) and murine Panc02 cells (kindly provided by Prof. V. Schmitz (University of Bonn, Germany)) were used. Human endothelial cells (endothelial cells, ECs) and vascular smooth muscle cells (VSMCs) were purchased from Promocell (Heidelberg, Germany). Cells were cultured in DMEM (Dulbecco’s Modified Eagle’s Medium; PAA Laboratories, Coelbe, Germany) supplemented with 15% FCS (fetal calf serum) maintained in 5% CO_2_ at 37°C as described. Human HGF was purchased from Peprotech (Hamburg, Germany). cMET inhibitor INC280 was kindly provided by Novartis Oncology (Basel, Switzerland) and dissolved in DMSO (*in vitro* use). For *in vivo* use, a stock solution with 0.5% methylcellulose and 0.1% Tween80 (Sigma-Aldrich, Munich, Germany) was prepared and further dissolved with water according to the manufacturer’s protocol. Mice received INC280 always via oral gavage around 1 p.m.. For hypoxia-mimicking, deferroxamine-mesylate (100 μM, DFX; Sigma-Aldrich) was used. Gemcitabine was purchased from our local pharmacy at the University of Regensburg. For *in vivo* use, mice received gemcitabine via i.p. injection in the afternoon.

To obtain cancer cell lines with acquired resistance to gemcitabine *in vitro*, MiaPaCa2 cells were treated with increasing doses of gemcitabine, starting from 10 nM up to 250 nM. Cells were subsequently named MiaPaCa2(G250).

### 3-(4,5-dimethylthiazol-2-yl)-2,5-diphenyltetrazolium (MTT) bromide assays

To evaluate cytotoxic effects of INC280, cells were seeded in 96-well plates (1 × 10^3^/well) and exposed to various concentrations of INC280. Experiments were performed in complete medium and upon serum-starvation ± HGF (50 ng/ml). We used the MTT assay to assess cell viability as described before [16,17].

### Migration assays

Migration assays with Boyden chambers (Becton Dickinson Bioscience, Heidelberg, Germany) were performed as reported [16,18]. HGF (50 ng/ml) or 15% FCS served as chemoattractant. After 24 hours, tumour cells were fixed and migrated cells stained (Diff-Quick reagent; Dade Behring, Newark, NJ). Migration assays with ECs and VSMCs were performed for 6 hours; cells that migrated through the filters were counted in four random fields and average numbers were calculated.

### Western blot analyses for activated signaling pathways and HIF-1α

Experiments were performed at a cellular density of 60% to 70%. Unless otherwise indicated, cells were incubated with increasing doses of INC280 (100, 500, 1000 nM) for 4 or 24 hours before stimulation with HGF (50 ng/ml) for 15 minutes. Whole-cell lysates were prepared as described before [17]. Membranes were sequentially probed with antibodies against phospho-Akt^Ser473^, Akt, phospho-ERK^Thr202/Tyr204^, ERK, phospho-cMET^Tyr1349^, cMET, phospho-FAK^Tyr925^, FAK (Cell Signaling, Beverly, MA), HIF-1α (Novus Biologicals, Littleton, CO) and β-actin (Santa Cruz Biotechnologies, Santa Cruz, CA). For detection of HIF-1α, MiaPaCa2(G250) cells were incubated for 24 hours with INC280 (500 nM) ± DFX (100 μM) as described [19]. Antibodies were detected by enhanced chemiluminescence (Amersham Bioscience, Piscataway, NJ).

### PCR analysis for MDR-1 expression

Expression of MDR-1 upon cMET inhibition with INC280 was determined by real-time PCR. Preparation of cDNA was performed as described (20). Selected primer pairs for PCR were as follows: MDR-1 (5-TGGCCTTaTTTTGTTGTTGGTG and 3-ATCATTGGCGAGCCTGGTAGTC) and 18S (5′-GTAACCCGTTGAACCCCATT and 3′-CCATCCAATCGGTAGTAGCG). Primers were optimized for MgCl_2_ and annealing, and PCR products were confirmed by gel electrophoresis. RT-PCR was done using the LightCycler system and Roche Fast-Start Light Cycler-Master Hybridization Probes master mix (Roche Diagnostics, Mannheim, Germany).

### Enzyme-linked immunosorbent assay for VEGF-A and PDGF-B

To determine changes in VEGF-A we used an ELISA kit specific for human VEGF-A (BioSource Europe, Nivelles, Belgium), as reported [20]. Pancreatic cancer cells were plated at 40-50% density and incubated with or without INC280 (500nM) and stimulated with DFX for 24 hours. Similar, PDGF-B was determined using an ELISA kit specific for human PDGF-B (Peprotech, Hamburg, Germany). Analyses of culture supernatants were performed according to the manufacturer’s protocol.

### Animal models

Experiments were approved by the Institutional Animal Care and Use Committee of the University of Regensburg and the regional authorities. In addition, experiments were conducted according to “Guidelines for the Welfare of Animals in Experimental Neoplasia” published by The United Kingdom Coordinating Committee on Cancer Research. Mice were housed in cages (n = 5 per unit) with tap water and food *ad libitum*. In addition, animals were assessed daily for tumour-associated symptoms and body weight was determined every other day. Effects of cMET inhibition with INC280 were first evaluated in an orthotopic pancreatic cancer model using human L3.6pl cancer cells. Briefly, 1 × 10^6^ L3.6pl cells were injected into the pancreatic tail of eight-week-old male athymic nude mice (BALB/c^nu/nu^, Charles River, Germany). Mice were randomized into 3 groups (n = 8–12/group) receiving either vehicle (controls) or INC280 (10 mg/kg/d or 20 mg/kg/d) by oral gavage based on previously reported dosing schedules [15]; treatment started 7 days after tumour cell inoculation. Mice were sacrificed after 28 days, tumours were excised, weighed, and incidence of macroscopically visible liver and lymph node metastases was determined. Subsequently, the effects of cMET inhibition with INC280 on growth of murine pancreatic cancer cells (Panc02) were confirmed in an orthotopic syngeneic model. Briefly, 2.5x10^5^ Panc02 pancreatic cancer cells were injected into the pancreatic tail of eight-week-old male C57BL/6 mice (Charles River, Germany). Mice were randomized into 2 groups (n = 7/group) and treatment with INC280 (10 mg/kg/d) was initiated on day 7 after tumour cell inoculation, according to the treatment schedule for orthotopic L3.6pl cells. Since tumours are more aggressive, the experiment was terminated on day 21 when mice in the control group showed signs of advanced disease. Tumours were excised, weighed, and macroscopically visible lymph node metastases and ascites were determined (Panc02 does not form liver metastases in our hands).

Since gemcitabine is the current chemotherapeutic standard for patients with pancreatic cancer, we next evaluated a gemcitabine dosing that delays, but does not abrogate tumour growth in our murine cancer model (similar to the situation in patients). For this purpose we used a subcutaneous syngeneic tumour model (Panc02, 1×10^5^ cells) with mice (n = 5/group) being treated with gemcitabine 50 or 100 mg/kg twice/week. Tumours from the higher dosing group were harvested at the end of the experiment and cMET expression determined by Western blotting. Based on the results from this model we next assessed the combination of INC280 (10 mg/kg/d) with gemcitabine (100 mg/kg twice/week) in the orthotopic syngeneic mouse model. To obtain a longer treatment period, we reduced the number of cells that were injected into the pancreatic tail to 1×10^5^. Mice were randomized into 4 groups (n = 7-9 mice/group) and therapy was initiated on day 7 after tumour cell implantation. On day 27 mice in the control group showed severe signs of tumour disease and, therefore, the experiment was terminated. Tumours were excised, weighed and occurrence of metastases was determined. In addition, tissue was harvested for immunohistochemical analyses.

To determine the efficacy of targeting cMET with INC280 on survival in advanced tumour stages, we first applied a subcutaneous tumour model. 1×10^5^ Panc02 cells were injected into the right flank of C57BL/6 mice and randomized to 4 groups (n = 10/group). To imitate the clinical setting in pancreatic cancer, the initial treatment in 20 mice was performed with gemcitabine (100 mg/kg twice/week) when tumours reached a size of 80 mm^3^. Upon progression to approximately 300 mm^3^, INC280 (10 mg/kg/d) was added to 10 mice pretreated with gemcitabine and 10 mice without any treatment. Mice were terminated when tumours reached a size of around 800 mm^3^. Since the local microenvironment has substantial impact on tumour growth and resistance, we finally performed a similar syngeneic orthotopic model. Again, 1×10^5^ Panc02 cells were injected into the pancreatic tail and mice were randomized to 4 groups (n = 10/group). Gemcitabine was given from day 10 (100 mg/kg twice/week) and INC280 (10 mg/kg/d) was added to the regime from day 20 after tumour cell injection, based on the results from the subcutaneous model. Mice were terminated as soon as they showed signs of tumour disease.

### Immunohistochemical analysis of vascularization, tumour cell proliferation and apoptosis

To determine CD31-positive vessel area, cryosections were obtained. Frozen tissue was fixed in cold acetone and chloroform, washed with PBS and exposed to antibodies against CD31 (1:50; Pharmingen, Germany), and respective secondary antibody (AlexaFluor 488; 1:200). Images were obtained in four different quadrants of each tumour section (2 mm inside the tumour- normal tissue interface) at 20× magnification [16]. Vessel area was determined as pixels/hpf using ImageJ version 1.46r (NIH, USA).

Ki67 staining was performed on paraffin sections. Briefly, slides were deparaffinized in xylene, followed by treatment with a graded series of alcohol washes [100, 95, 70% ethanol/ddH_2_O (v/v)], rehydration in citrate buffer (pH6; Merck, Darmstadt, Germany), and blocking against endogenous peroxidase with H_2_O_2_. Slides were incubated with Ki67 primary antibody (1:100; abcam, Cambridge, UK) at 4°C overnight. After washing with TBS, secondary antibody (Santa Cruz Biotechnologies, Santa Cruz, CA) was added to tissue sections followed by incubation with diaminobenzidine (Biozol, Eching, Germany). Negative controls were performed by omitting the primary antibody. Ki67 positive cells were counted in four fields per tumour section at 20× magnification and the average was calculated.

A terminal deoxynucleotidyl transferase-mediated nick-end labeling detection kit (TUNEL; Promega Corp., Mannheim, Germany) was used to detect cell apoptosis [20]. Four fields at 20× magnification were selected at the proliferation front in each tumour, and TUNEL positive cells were counted; an average value from these results was calculated.

### Statistical analysis

Statistical analyses were done using SigmaStat (Version 3.0). Results of *in vivo* experiments were analyzed for significant outliers using the Grubb’s test (www.graphpad.com). Tumour-associated variables of *in vivo* experiments were tested for statistical significance using the Mann–Whitney *U* test for nonparametric data or ANOVA followed by Tukey’s multiple comparison test for more than two groups. The two-sided Student’s *t*-test was applied for analysis of *in vitro* data. All results are expressed as the mean ± standard error of the mean (SEM).

## Results

### Effect of cMET inhibition on pancreatic cancer cells *in vitro*

**Figure 1:**
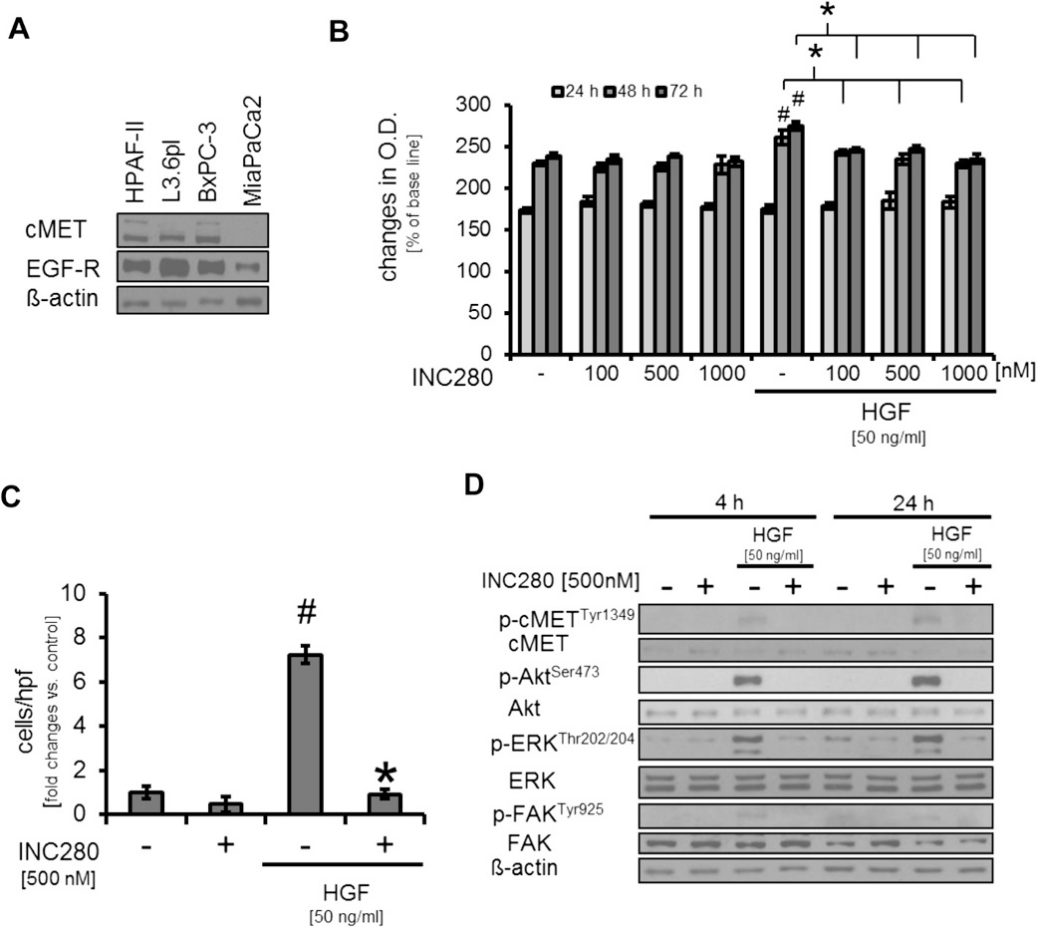
**Effects of targeting cMET on pancreatic cancer cells. A)** cMET expression was detectable in HPAF-II, L3.6pl, BxPC3 but not in parental MiaPaCa2 pancreatic cancer cell lines. **B)** Incubation of HPAF-II cells with the cMET inhibitor INC280 has no effect on constitutive growth. When cells were stimulated with HGF, a significant growth increase was observed (^#^*P* < 0.05). This was abrogated by cMET inhibition with INC280 (*P < 0.05). **C)** HGF induces cancer cell motility (^#^*P* < 0.05) that can be efficiently blocked by INC280 (*P < 0.05). Constitutive motility remains unaffected. **D)** Treatment with INC280 disrupts HGF-mediated phosphorylation of Akt, ERK and FAK after 4 and 24 hours of treatment. Results in 1B-1D are shown for HPAF-II; similar results were obtained from L3.6pl and Panc02. Bars = SEM.

First, the expression of cMET as the INC280 target was assessed in human pancreatic cancer cell lines. Results showed that cMET is expressed in HPAF-II, BxPC3 and L3.6pl cells, whereas MiaPaCa2 did not show detectable expression (Figure 1A). Next, the effects of targeting cMET with INC280 on growth of pancreatic cancer cell lines were determined *in vitro*. Using MTT assays a slight, but significant, dose-dependent growth inhibition of HPAF-II was found only when cells were stimulated with HGF (Figure 1B). Subsequently, migration assays showed that cMET blockade significantly impairs HGF-induced motility but had no effect on constitutive migration in HPAF-II cells (Figure 1C). Last, we determined the impact of INC280 on activation of oncogenic signaling pathways. Incubation of HPAF-II cells with INC280 for 4 hours or 24 hours did not affect constitutive Akt, ERK or FAK phosphorylation. While no constitutive cMET phosphorylation was observed upon these conditions, stimulation with HGF for 15 minutes led to phosphorylation of cMET, Akt, ERK and FAK. Pretreatment with INC280 completely abrogated this effect (Figure 1D). The described observations were subsequently confirmed in the L3.6pl human pancreatic cancer cell line (Additional file 1: Figure S1A-C); FAK phosphorylation was not detectable in L3.6pl and therefore not shown. In addition, we used the murine pancreatic cancer cell line Panc02 to confirm these results (data not shown). Taken together, our results show that treatment with INC280 efficiently abrogates HGF-induced motility and oncogenic signaling in pancreatic cancer cell lines *in vitro*.

### Impact of targeting cMET on gemcitabine-resistant cancer cells *in vitro*

**Figure 2:**
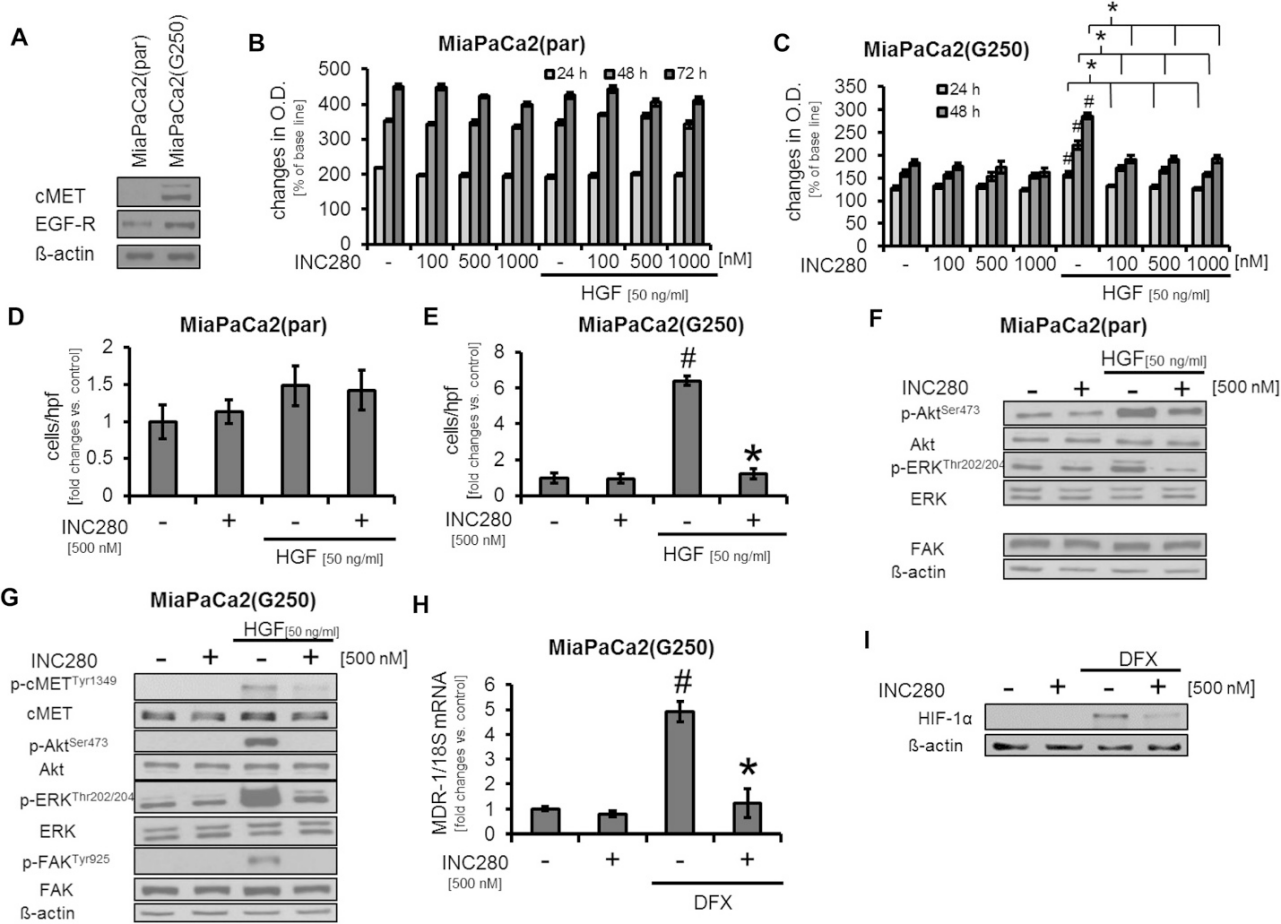
**Targeting cMET in parental MiaPaCa2 (MiaPaCa2(par)) and resistant MiaPaCa2 (MiaPaCa2(G250)) pancreatic cancer cell lines. A)** In MiaPaCa2(G250) cells, cMET expression was detectable whereas no cMET was found in MiaPaCa2(par). **B)** MiaPaCa2(par) showed little response to cMET inhibition with INC280 even when cells were stimulated with HGF (50 ng/ml). **C)** Growth of MiaPaCa2(G250) was significantly improved when cells were incubated with HGF (^#^*P* < 0.05). cMET inhibition with INC280 inhibited this growth induction (*P < 0.05). **D)** Migration of MiaPaCa2(par) was increased when cells were stimulated with HGF (not significant); cMET inhibition did not affect constitutive or HGF-induced motility in these cells. **E)** In MiaPaCa2(G250) stimulation with HGF led to a significant increase in cancer cell motility (^#^*P* < 0.05), which was abrogated by targeting cMET with INC280 (*P < 0.05). **F)** In MiaPaCa2(par) stimulation with HGF led to weak induction of Akt and ERK phosphorylation. This was diminished by treatment with INC280. No effects on constitutive signaling were observed. **G)** HGF showed strong induction of Akt, ERK and FAK phosphorylation in MiaPaCa2(G250). Targeting cMET impairs effects on HGF-induced activation of signaling intermediates. **H)** Mimicking hypoxic conditions with DFX (100 μM) significantly induced MDR-1 mRNA expression in MiaPaCa2(G250) (^#^*P* < 0.05) and cMET inhibition with INC280 abrogated this (*P < 0.05). MDR-1 mRNA was not detectable in MiaPaCa2(par). Bars = SEM **I)** INC280 impairs DFX induced HIF-1α expression in MiaPaCa2(G250) cells.

**Figure 3:**
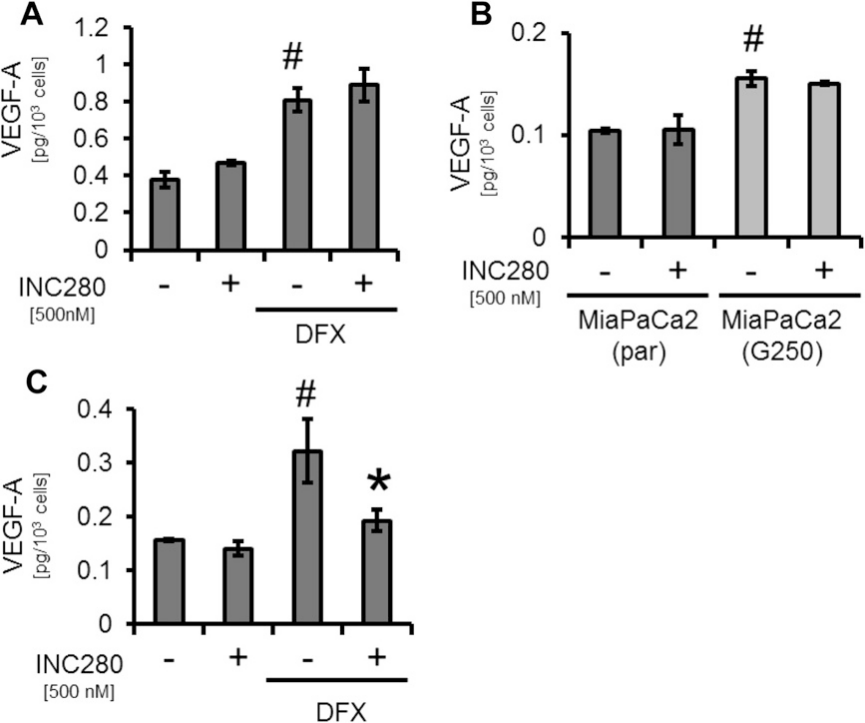
**Effects on VEGF-A secretion by cMET inhibition. A)** cMET inhibition had no effect on constitutive or DFX-induced VEGF-A secretion by HPAF-II pancreatic cancer cells (^#^*P* < 0.05). **B)** MiaPaCa2(G250) showed a significant increase in VEGF-A production compared to MiaPaCa2(par) (^#^*P* < 0.05). However, INC280 has no effect on constitutive VEGF-A secretion in either MiaPaCa2(par) or gemcitabine-resistant MiaPaCa2(G250). **C)** Hypoxia-mimicking DFX significantly induced VEGF-A secretion from MiaPaCa2(G250) (^#^*P* < 0.05). Targeting cMET significantly impaired this effect (*P < 0.05). Bars = SEM.

The pancreatic cancer cell line MiaPaCa2 confers a certain resistance against gemcitabine which is the accepted standard for systemic treatment in pancreatic cancer [21,22]. To further enforce this resistance, MiaPaCa2 cells were treated with increasing doses of gemcitabine starting from 10 nM up to 250 nM. Interestingly, although no cMET expression was detectable in the native cell line, MiaPaCa2 cells treated with gemcitabine showed strong expression of cMET and a slight increase in EGFR expression (Figure 2A). Subsequently, we compared the properties of native (parental) MiaPaCa2 cells (also named MiaPaCa2(par)) with those pretreated with gemcitabine (named MiaPaCa2(G250)) with regards to cMET inhibition with INC280. In MTT assays, MiaPaCa2(par) did not respond to HGF stimulation and thus to cMET inhibition with INC280, as one would expect because of the missing cMET receptor (Figure 2B). In contrast, HGF strongly induced growth of MiaPaCa2(G250) and INC280 significantly impaired this (Figure 2C). Migration assays showed that HGF tended to increase cancer cell motility in MiaPaCa2(par), but INC280 did not affect either constitutive or HGF-induced motility (Figure 2D). In MiaPaCa2(G250) HGF led to a more than 6-fold increase in motility which was abrogated by cMET blockade (Figure 2E). Finally, Western blotting from MiaPaCa2(par) showed only modest phosphorylation of ERK and Akt upon HGF stimulation and INC280 had only minor effects on Akt but impaired ERK phosphorylation (Figure 2F). In MiaPaCa2(G250), a strong phosphorylation of Akt, ERK and FAK upon stimulation with HGF was detected, which was strongly inhibited by INC280 (Figure 2G). Next, we assessed the expression of MDR-1 as a known mediator of multidrug-resistance [23,24]. Interestingly, MiaPaCa2(par) do not express MDR-1 mRNA, whereas MiaPaCa2(G250) showed MDR-1 mRNA expression which was strongly induced upon incubation with hypoxia-mimicking DFX. Treatment with INC280 significantly reduced MDR-1 mRNA expression in MiaPaCa2(G250) cells (Figure 2H). Searching for the mechanism of MDR-1 regulation, we found that expression of hypoxia-induced HIF-1α, a major regulator of MDR-1 and stromal factors such as VEGF-A, is impaired by cMET inhibition in MiaPaCa2(G250) (Figure 3I). In conclusion, these results indicate that cMET expression is involved in resistance to gemcitabine and INC280 effectively inhibits HGF-induced effects in gemcitabine-resistant pancreatic cancer cell lines.

### Modulation of stromal factors in pancreatic cancer cell lines *in vitro*

Since cancer cells are a major source of factors that influence the microenvironment [25], we next determined whether cMET inhibition affects secretion of VEGF-A and PDGF-B in cancer cell lines. Using ELISAs for VEGF-A and PDGF-B, we found no effect of targeting cMET on secretion of both in HPAF-II and L3.6pl even when cells were stimulated with hypoxia-mimicking DFX (Figure 3A and Additional file 2: Figure S2B for HPAF-II, Additional file 2: Figure S2A and 2C for L3.6pl). Interestingly, we detected an increase in VEGF-A and PDGF-B secretion in MiaPaCa2(G250) cells compared to MiaPaCa2(par). Nonetheless, INC280 had no effect on constitutive secretion of both factors in MiaPaCa2(par) or in MiaPaCa2(G250), suggesting that other cMET-independent mechanisms are involved in this up-regulation (Figure 3B, Additional file 2: Figure S2D). Finally, we assessed the effect of cMET inhibition on hypoxia-induced VEGF-A secretion in MiaPaCa2(G250) since INC280 led to inhibition of HIF-1α in these cells. Results showed a strong DFX-induced increase in VEGF-A secretion that was significantly impaired by cMET blockade (Figure 3C). Regarding PDGF-B, incubation with DFX also led to a significant induction of protein secretion, but this was not affected by cMET blockade (Additional file 2: Figure S2E). Together, these results suggest that targeting cMET in pancreatic cancer cell lines has no effect on VEGF-A and PDGF-B secretion. However, the secretion of VEGF-A in gemcitabine-resistant cells might be affected by cMET inhibition via inhibition of HIF-1α.

### Targeting cMET in stromal components (ECs, VSMCs) *in vitro*

**Figure 4:**
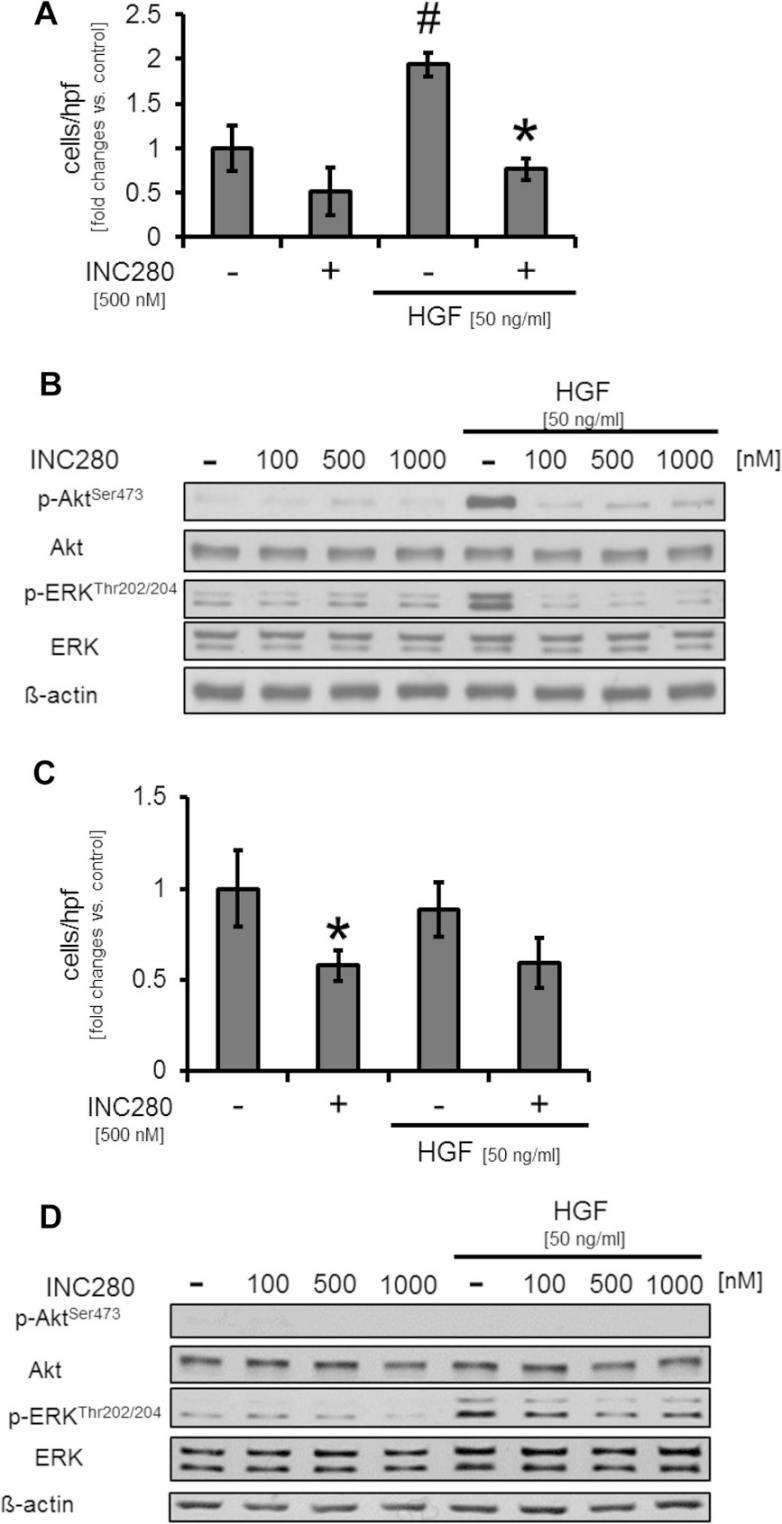
**Effects of cMET inhibition on endothelial cells (ECs) and vascular smooth muscle cells (VSMCs). A)** HGF significantly induced motility of EC *in vitro (*^#^*P* < 0.05); INC280 impaired this effect (*P < 0.05). **B)** cMET inhibition had no effect on constitutive activation of signaling pathways in ECs. However, HGF strongly induces Akt and ERK phosphorylation which can efficiently be inhibited by INC280. **C)** Stimulation with HGF had no effect on motility of VSMCs. However, INC280 impaired constitutive motility of these cells *in vitro* (*P < 0.05). **D)** In VSMCs, HGF had minor effects on Akt and ERK phosphorylation *in vitro*. INC280 impairs the effect of HGF, but has no effect on constitutive pathway activation. Bars = SEM.

Pancreatic cancer is characterized by a strong stromal reaction. Therefore, we subsequently examined the effects of cMET inhibition on ECs and VSMCs. MTT assays in ECs under serum-starved conditions and stimulation with HGF, showed a slight but significant increase in growth that was diminished by INC280 (Additional file 3: Figure S3B). No effect upon constitutive conditions was observed (Additional file 3: Figure S3A). EC motility was significantly increased upon incubation with HGF, which was strongly reduced by INC280 (Figure 4A). Regarding activation of signaling pathways, treatment with INC280 strongly inhibited HGF-induced activation of Akt and ERK whereas no effects on constitutive Akt and ERK phosphorylation were found (Figure 4B). Taken together, these results show that INC280 affects ECs only when these cells are stimulated with HGF.

Next we analyzed the impact of INC280 on VSMCs. MTT assays showed a dose-dependent inhibition of VSMC growth starting from INC280 (100nM) after 72 hours of incubation (Additional file 3: Figure S3C). In contrast to ECs, stimulation with HGF upon serum-starved conditions had no effect on VSMC growth and, accordingly, INC280 did not have a further growth inhibitory effect in MTT assays (Additional file 3: Figure S3D). Motility upon incubation with HGF in VSMCs was not induced, but targeting cMET with INC280 led to a significant inhibition of constitutive migration (Figure 4C). Finally, Western blotting did not show a substantial effect of INC280 on constitutive Akt phosphorylation and only a minor impact on ERK phosphorylation in VSMCs (Figure 4D). These results indicate that HGF does not affect VSMCs and cMET inhibition with INC280, therefore, has only minor effects on these cells.

### Targeting cMET impairs tumour growth *in vivo*

**Figure 5:**
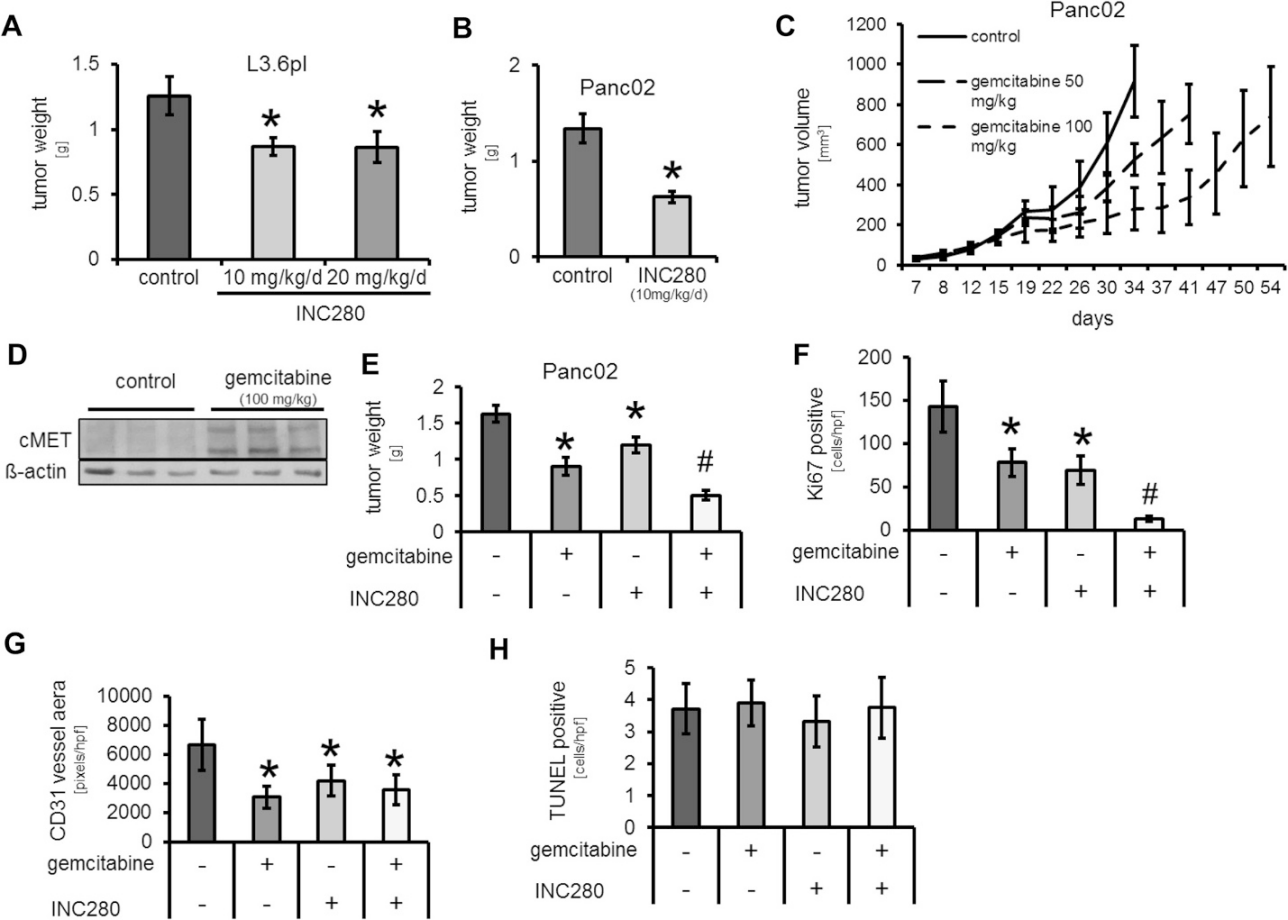
**Targeting cMET with INC280*in vivo*. A)** cMET inhibition significantly impaired tumour growth in the orthotopic xenogeneic tumour model using L3.6pl human pancreatic cancer cell line. Increasing the dose to 20 mg/kg/d did not improve growth-inhibitory effects (*P < 0.05). **B)** Similar growth inhibition was observed in the orthotopic syngeneic model with murine Panc02 cells (*P < 0.05). **C)** In the subcutaneous syngeneic tumour model, treatment with gemcitabine (50 and 100 mg/kg twice/week) led to a transient deceleration of tumour growth, however, even when gemcitabine therapy was continued, tumours soon reentered the exponential growth phase. **D)** Expression of cMET in tumours treated with gemcitabine (100 mg/kg twice/week) was markedly increased as compared to untreated controls. **E)** INC280 and gemcitabine led to significant inhibition of tumour growth in the orthotopic syngeneic model (*P < 0.05 vs. control). Combination of both substances substantially enhanced this effect (^#^*P* < 0.01 vs. control and ^#^*P* < 0.05 vs. gemcitabine and INC280 alone). **F)** INC280 and gemcitabine significantly reduced tumour cell proliferation (Ki67) *in vivo*. Combination of both substances further increased the anti-proliferative capacity (^#^*P* < 0.01 vs. control and ^#^*P* < 0.05 vs. gemcitabine and INC280 alone). **G)** Significant reduction of tumour angiogenesis was observed upon either single agent or combination therapy, as determined by CD31 positive vessel area (*P < 0.05 vs. control). **H)** No effect on tumour cell apoptosis was detected by TUNEL staining.

Our results so far suggest that cMET inhibition with INC280 might be effective against pancreatic cancer cells. To further address this issue we used an orthotopic xenograft model with metastatic L3.6pl pancreatic cancer cells. To elucidate a potential dose-dependent effect, treatment with INC280 (10 and 20 mg/kg/d) was initiated 7 days after tumour cell inoculation. Results showed a significant reduction of final tumour weight after 28 days in both treatment groups, compared to control (Figure 5A). However, no difference between the 10 mg/kg/d and the 20 mg/kg/d group was noted. Therefore, 10 mg/kg/d was selected as the effective dose in follow-up experiments. Notably, we also found a trend towards reduced liver metastases upon cMET blockade, although this did not reach statistical significance (Table 1). Moreover, enlarged lymph nodes were detected in 8/12 (66%) mice in the control group, but only 3/10 (30%) in the 10 mg/kg/d group and 2/8 (25%) in the 20 mg/kg/d group (Table 1); these results only describe a trend, since statistical significance was not reached. Nonetheless, this is of particular importance since lymph nodes are the primary site of metastasis in pancreatic cancer. To confirm the growth inhibitory effects of INC280, we subsequently used the syngeneic orthotopic model with murine Panc02 cells (2.5×10^5^ cells). Results again showed that cMET inhibition (10 mg/kg/d) significantly impairs tumour growth on day 21 (Figure 5B). In this model we also found a trend towards reduction of lymph node metastases similar to the orthotopic xenogeneic model (Table 1). We were not able to examine liver metastases in the Panc02 model since these cells do not form liver metastases in our experience. It should be noted that none of the mice treated with INC280 showed ascites formation (Table 1). In summary, these results clearly demonstrate that targeting cMET with INC280 impairs tumour growth and metastases *in vivo*.

**Table 1:** **Metastases formation in the orthotopic pancreatic cancer xenogeneic and syngeneic model**

L3.6pl orthotopic model^1^				
	Liver metastases		LN metastases	
	Positive	Negative	Positive	Negative
Control (n = 12)	8 (66.7%)	4 (33.3%)	8 (66.7%)	4 (33.3%)
INC280 10 mg/kg/d (n = 10)	5 (50%)	5 (50%)	3 (30%)	7 (70%)
INC280 20 mg/kg/d (n = 8)	4 (50%)	4 (50%)	2 (25%)	6 (75%)
**Panc02 orthotopic model** ^**2**^				
	LN metastases		Ascites	
	Positive	Negative	Positive	Negative
Control (n = 7)	4 (57.1%)	3 (42.9%)	4 (57.1%)	3 (42.9%)
INC280 10 mg/kg/d (n = 7)	0	7 (100%)	0	7 (100%)
**Panc02 orthotopic model** ^**2**^				
	LN metastases		Ascites	
	Positive	Negative	Positive	Negative
Control (n = 9)	6 (66.7%)	3 (33.3%)	3 (33.3%)	6 (66.7%)
INC280 (n = 7)	1 (14.3%)	6 (85.7%)	1 (14.3%)	6 (85.7%)
gemcitabine (n = 8)	2 (25%)	6 (75%)	2 (25%)	6 (75%)
INC280 + gemcitabine (n = 8)	1 (12.5%)	7 (87.5%)*	0 (0%)	8 (100%)

^1^does not form ascites in our hands.

^2^does not form liver metastases in our hands.

*P < 0.05 vs. control.

### Combination of cMET inhibition with gemcitabine treatment *in vivo*

Since gemcitabine is the standard treatment for pancreatic cancer patients, we next addressed the issue of combining INC280 with gemcitabine *in vivo*. In accordance to the situation in patients, we first defined a dosing for gemcitabine that has only limited therapeutic efficacy using a subcutaneous syngeneic tumour model (Panc02). Results showed that 50 and 100 mg/kg gemcitabine administered twice/week significantly delays, but does not abrogate, tumour growth in the Panc02 model (Figure 5C). In addition, Western blotting from these tumours revealed an up-regulation of cMET expression upon gemcitabine treatment (Figure 5D). In further experiments, we chose to use gemcitabine 100 mg/kg twice/week in combination with INC280 (10 mg/kg/d). The efficacy of this combination was first assessed in the orthotopic syngeneic model (Panc02, 1×10^5^ cells). Simultaneous treatment with INC280 and gemcitabine was initiated 7 days after tumour cell implantation and went on for 20 days. No treatment-associated side effects (e.g. weight loss) were observed (data not shown). After 27 days results revealed a significant reduction of tumour weight in all treatment groups; the combination treatment was most effective showing significant tumour reduction versus both the control and single agent therapy groups (Figure 5E). Immunohistochemical work-up revealed a significant inhibition of tumour cell proliferation (Ki67 staining) in all treatment groups, but the combination treatment was clearly most effective (Figure 5F). Regarding tumour vascularization, CD31 staining revealed a significant reduction in all treatment groups compared to controls, although there was no difference between the different treatment groups (Figure 5G). No effect on tumour cell apoptosis was observed (Figure 5H). Metastases formation was also impaired in this model with only 1/8 mice showing enlarged lymph nodes and no ascites with combined INC280 and gemcitabine treatment (Table 1). These results clearly show the potential therapeutic benefits of combining cMET inhibition with gemcitabine treatment.

### Inhibition of cMET prolongs survival in combination with gemcitabine *in vivo*

**Figure 6:**
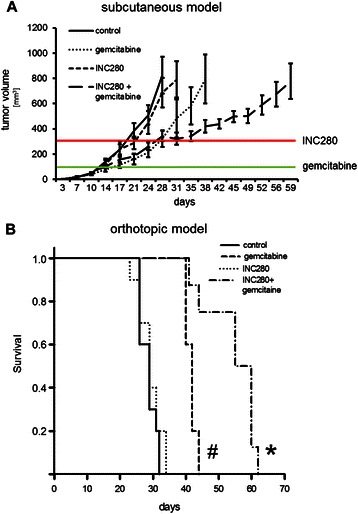
**Impact of gemcitabine and INC280 combination therapy in advanced tumour stages. A)** In the subcutaneous syngeneic mouse model, treatment with gemcitabine was initiated when tumours reached a size of 80–100 mm^3^ (green line). INC280 treatment was started when tumour size exceeded 300 mm^3^ (red line). Gemcitabine alone led to a delay in tumour growth; Combination of gemcitabine with cMET inhibition significantly improved tumour growth inhibition. **B)** In the orthotopic syngeneic tumour model, survival of mice was significantly prolonged with gemcitabine therapy alone (^#^*P* < 0.05 vs. control and vs. INC280). A significant survival improvement was observed when gemcitabine was combined with cMET inhibition (*P < 0.001 vs. control and *P* < 0.05 vs. gemcitabine).

The clinical situation shows that more than 80% of patients present with an advanced tumour stage and are initially treated with systemic chemotherapy. We addressed this issue first in a subcutaneous tumour model (Panc02). Gemcitabine treatment (100 mg/kg twice/week) was started when tumours reached a size of 80–100 mm^3^ (Figure 6A, green line). From our initial experiments with gemcitabine in the subcutaneous tumour model we knew that this leads to delayed tumour growth up to a size of approximately 300 mm^3^ (Figure 5C). Therefore, we defined tumours >300 mm^3^ as clinically progressing, and used this threshold to add INC280 as “second line” therapy (Figure 6A, red line). Mice were sacrificed when tumours reached a size of 800 mm^3^. Results showed that gemcitabine leads to growth delay *in vivo* as expected and delayed initiation of INC280 treatment alone has no effect on tumour growth. In comparison, combined INC280 and gemcitabine treatment was the most effective therapy against tumour progression (Figure 6A). Since the microenvironment has significant impact on tumour growth and resistance to anti-neoplastic therapies, we confirmed these results in the orthotopic syngeneic model (Panc02). In accordance with the subcutaneous model experiments, we initiated gemcitabine (100 mg/kg twice/week) treatment on day 10 and added INC280 (10 mg/kg/d) on day 20. Results from this model showed that INC280 alone has no effect on advanced tumour growth, and gemcitabine significantly improves survival as expected. Consistent with the subcutaneous model results, the addition of the cMET inhibitor INC280 to tumours pretreated with gemcitabine was the most efficient therapy (Figure 6B). These results strongly support the idea of adding cMET inhibition to gemcitabine treatment, even when the pancreatic cancer is in advanced stages.

## Discussion

Pancreatic cancer is still one of the most devastating tumour entities in humans and surgical resection provides the only curative option. However, most patients present in stages where surgery is not an option. Therefore, novel therapeutic opportunities are desperately needed to improve the prognosis. Within the current study we assess the effects of targeting cMET by using a novel orally available ATP-competitive inhibitor in pancreatic cancer models. Our results show that cMET inhibition impairs activation of HGF-induced oncogenic signaling intermediates in human and murine pancreatic cancer cell lines. Moreover, treatment with the cMET inhibitor reduces tumour growth *in vivo* and prolongs survival of mice most effectively when used in combination with gemcitabine. Taken together, our results suggest that targeting cMET might be a novel way to improve outcome of patients with pancreatic cancer.

Low level expression of cMET and HGF is known in the exocrine pancreas. However, when proceeding to PanIN or even invasive ductal adenocarcinomas, expression of both cMET and HGF greatly increases [11,26,27]. Several studies have linked activation of cMET signaling to phosphorylation of intracellular signaling cascades such as PI3K/Akt, MAPK/ERK or FAK in pancreatic cancer models, leading to tumour cell invasiveness, motility and resistance to gemcitabine therapy [12,13,28-31]. Within the current study, we confirm that treatment of cancer cell lines with the cMET inhibitor INC280 significantly impairs HGF-induced growth and motility of tumour cells, at least in part via inhibition of Akt, ERK and FAK phosphorylation. Of note, there was no effect detected on growth, motility and signaling intermediates in cancer cell lines under constitutive conditions. This is of particular importance since the stroma is known to be a major source of HGF and Ide *et al*. showed that HGF expression in the stroma of pancreatic cancer specimens is associated with poor prognosis in these patients [32]. Hence, phosphorylation of oncogenic signaling cascades in pancreatic cancer cells via paracrine activation of the cMET receptor might contribute to pancreatic cancer aggressiveness. Therefore, it seems logical that targeting cMET with INC280 would negate this mechanism.

A major issue in pancreatic cancer therapy is its resistance to almost every systemic therapy. Gemcitabine, which was for many years considered to be the standard therapy for pancreatic cancer, only has response rates between 5.6 and 13.3% [33]. Novel therapeutic regimes such as FOLFIRINOX show response rates only around 30% and come at a cost of high toxicity in more than 50% of patients [4]. Previous studies have connected the resistance of pancreatic cancer cell lines to gemcitabine, with alterations in EMT (epithelial-to-mesenchymal transition) that include the cMET receptor [34]. Moreover, a report by Li and coworkers defined cMET as a marker for pancreatic cancer stem cells (CSC) with a high self-renewal capacity [35]. Recently, Hage and colleagues demonstrated that treatment with cabozantinib, a dual inhibitor of cMET and VEGFR-2, increases the efficacy of gemcitabine, even when cells were resistant to this agent [12]. In our experiments MiaPaCa2 pancreatic cancer cells also show an increase in cMET expression when resistant to gemcitabine (MiaPaCa2(G250)). Moreover, the cMET receptor seems to be functionally active since incubation with HGF induces growth, motility and phosphorylation of intracellular signaling cascades in these cells. Interestingly, MiaPaCa2(G250) cells that are resistant to gemcitabine express MDR-1 mRNA which is in contrast to normal MiaPaCa2(par); MDR-1 is up-regulated in MiaPaCa2(G250) cells under hypoxic conditions. This is of particular importance since expression of MDR-1 and its encoding protein P-gp have been implicated in reduced drug up-take, thereby mediating resistance to chemotherapy [36,37]. Regulation of MDR-1 has been linked to HIF-1 activation/stabilization and previous reports indicate that cMET blockade impairs this transcription factor in cancer cells [38,39]. Indeed, we found a reduction of HIF-1α upon cMET inhibition in MiaPaCa2(G250) cells. In conclusion, treatment with the cMET inhibitor INC280 impairs both hypoxia-induced MDR-1 expression and HGF-mediated effects on growth, motility and signaling intermediates in gemcitabine resistant cancer cells *in vitro*. One might speculate that treatment with gemcitabine leads to selection of cancer cells (potentially cancer stem cells) with high cMET expression, thereby providing a rational for the use of cMET inhibitors in pancreatic cancer at least when tumours become gemcitabine resistant.

We used syngeneic orthotopic pancreatic cancer models to further evaluate our findings. The advantage of these models is the existence of a functional immune system in the host, and the presence of a proper local microenvironment [40]. As predicted from our *in vitro* data, combination of gemcitabine with the cMET inhibitor INC280 was far superior to single agent therapy in terms of tumour inhibition in the orthotopic model. Interestingly, this effect correlated to inhibition of tumour cell proliferation, with no difference in tumour cell apoptosis detected; these results are consistent with growth inhibition caused by the combination of gemcitabine and cMET inhibitor previously reported by Li *et al*. [35]. However, in contrast to our study, Li and coworkers used immunocompromised NOD/SCID mice with primary human pancreatic adenocarcinoma cells. Regarding apoptosis, a study by Hage and coworkers described a significant increase *in vitro* when pancreatic cancer cell lines were treated with a cMET inhibitor in combination with gemcitabine [12]. However, these results were not confirmed by *in vivo* experiments. Taken together, our study substantiates the rational for the use of cMET inhibitors in combination to gemcitabine in patients with pancreatic cancer.

The clinical situation shows that most patients present in an advanced stage of disease, necessitating the use of models that address this situation. We were particularly optimistic about the strategy to use cMET inhibitors since we found that tumours of mice treated with gemcitabine strongly up-regulate the cMET receptor. To mimic the clinical situation, we chose to apply INC280 at an advanced tumour stage in the syngeneic orthotopic survival model. Importantly, adding the cMET inhibitor at a progressive tumour growth stage, together with gemcitabine therapy, produced significantly prolonged survival in mice. These results are in line with a recently published study by Avan and colleagues showing a significant improvement in survival when combining gemcitabine with the ATP-competitive cMET inhibitor crizotinib in mice bearing primary pancreatic ductal adenocarcinoma specimen [28]. In contrast to Avan and coworkers who simultaneously started treatment with the inhibitor and gemcitabine 5 days after tumour implantation, we selected an even later time point to begin INC280 treatment (20 days after tumour cell inoculation), and initiated gemcitabine earlier at 10 days. Since gemcitabine remains a “first-line” therapy, and other treatments are typically considered after the tumour shows resistance, we feel the experimental model we used is closer to the clinical situation.

## Conclusion

In summary, the present study shows that targeting cMET may lead to an effective inhibition of tumour growth in pancreatic cancer, even in advanced tumour stages. Particularly, inhibition of gemcitabine-resistance in tumour cells by cMET inhibitors may improve current anti-neoplastic therapy strategies for the treatment of pancreatic cancer patients.

## Additional files

Additional file 1: Figure S1. Effects of targeting cMET on L3.6pl pancreatic cancer cells. A) Incubation of L3.6pl cells with the cMET inhibitor INC280 has no effect on constitutive growth. When cells were stimulated with HGF, a significant improvement of growth was observed (#*P<0.05*). This was abrogated by cMET inhibition with INC280 (**P*<0.05). B) HGF induces cancer cell motility (#*P*<0.05) that can efficiently be blocked by INC280 (**P*<0.05). Constitutive motility remains unaffected. C) Treatment with INC280 disrupts HGF-mediated phosphorylation of Akt and ERK after 4 and 24 hours of treatment. Bars=SEM.
Additional file 2: Figure S2. Effects of targeting cMET on VEGF-A and PDGF-B secretion from cancer cells. A) Hypoxia led to a significant increase in VEGF-A secretion from L3.6pl cancer cells (#*P*<0.05). INC280 did not affect this. B) Induction with DFX led to a significant increase in PDGF-B secretion from HPAF-II pancreatic cancer cells (#*P*<0.05). Targeting cMET had no impact on this. C) Similar, DFX led to increase in PDGF-B secretion from L3.6pl and INC280 did not reduce this *in vitro* (#*P*<0.05). D) Gemcitabine resistant MiaPaCa2(G250) showed a higher secretion of PDGF-B compared to regular MiaPaCa2(par) (#*P*<0.05). cMET inhibition had no effect on this *in vitro*. E) Incubation with DFX led to a significant increase in PDGF-B secretion from MiaPaCa2(G250) but INC280 did not affect this increase (#*P*<0.05). Bars=SE.
Additional file 3: Figure S3. Effects of targeting cMET on ECs and VSMCs. A) No effect of targeting cMET with INC280 on ECs was detected upon constitutive conditions. B) HGF induces growth of ECs after 48 and 72 hours of incubation (#*P*<0.05). Incubation with INC280 impairs this (**P*<0.05). C) In VSMCs, INC280 led to inhibition of constitutive growth upon constitutive conditions (**P*<0.05). D) Upon serum-starved conditions, no effect on constitutive growth was detectable. Similar, stimulation of VSMCs with HGF did not affect *in vit*ro growth. Bars = SE.

## Abbreviations

MAPK: Mitogen-activated protein kinase
ERK: Extracellular signal-regulated kinase
DMEM: Dulbecco’s modified Eagle’s medium
FCS: Fetal calf serum
HGF: Hepatocyte growth factor
PDGF: Platelet derived growth factor
VEGF: Vascular endothelial growth factor
FAK: Focal adhesion kinase
MTT: 3-(4,5-dimethylthiazol-2-yl)-2,5-diphenyltetrazolium (MTT) bromide assays
ECs: Endothelial cells
VSMCs: Vascular smooth muscle cells
DFX: Desferroxamine
hpf: High-power field

## References

[1] Malvezzi M, Bertuccio P, Levi F, La Vecchia C, Negri E (2013). European cancer mortality predictions for the year 2013. Ann Oncol.

[2] Siegel R, Naishadham D, Jemal A (2013). Cancer statistics, 2013. CA Cancer J Clin.

[3] Stathis A, Moore MJ (2010). Advanced pancreatic carcinoma: current treatment and future challenges. Nat Rev Clin Oncol.

[4] Conroy T, Desseigne F, Ychou M, Bouche O, Guimbaud R, Becouarn Y (2011). FOLFIRINOX versus gemcitabine for metastatic pancreatic cancer. N Engl J Med.

[5] Bladt F, Riethmacher D, Isenmann S, Aguzzi A, Birchmeier C (1995). Essential role for the c-met receptor in the migration of myogenic precursor cells into the limb bud. Nature.

[6] Chmielowiec J, Borowiak M, Morkel M, Stradal T, Munz B, Werner S (2007). c-Met is essential for wound healing in the skin. J Cell Biol.

[7] Huh CG, Factor VM, Sanchez A, Uchida K, Conner EA, Thorgeirsson SS (2004). Hepatocyte growth factor/c-met signaling pathway is required for efficient liver regeneration and repair. Proc Natl Acad Sci U S A.

[8] Blumenschein GR, Mills GB, Gonzalez-Angulo AM (2012). Targeting the hepatocyte growth factor-cMET axis in cancer therapy. J Clin Oncol.

[9] Gherardi E, Birchmeier W, Birchmeier C, Vande Woude G (2012). Targeting MET in cancer: rationale and progress. Nat Rev Cancer.

[10] Zhu GH, Huang C, Qiu ZJ, Liu J, Zhang ZH, Zhao N (2011). Expression and prognostic significance of CD151, c-Met, and integrin alpha3/alpha6 in pancreatic ductal adenocarcinoma. Dig Dis Sci.

[11] Park JK, Kim MA, Ryu JK, Yoon YB, Kim SW, Han HS (2012). Postoperative prognostic predictors of pancreatic ductal adenocarcinoma: clinical analysis and immunoprofile on tissue microarrays. Ann Surg Oncol.

[12] Hage C, Rausch V, Giese N, Giese T, Schonsiegel F, Labsch S (2013). The novel c-Met inhibitor cabozantinib overcomes gemcitabine resistance and stem cell signaling in pancreatic cancer. Cell Death Dis.

[13] Bauer TW, Somcio RJ, Fan F, Liu W, Johnson M, Lesslie DP (2006). Regulatory role of c-Met in insulin-like growth factor-I receptor-mediated migration and invasion of human pancreatic carcinoma cells. Mol Cancer Ther.

[14] Hill KS, Gaziova I, Harrigal L, Guerra YA, Qiu S, Sastry SK (2012). Met receptor tyrosine kinase signaling induces secretion of the angiogenic chemokine interleukin-8/CXCL8 in pancreatic cancer. PLoS One.

[15] Liu X, Wang Q, Yang G, Marando C, Koblish HK, Hall LM (2011). A novel kinase inhibitor, INCB28060, blocks c-MET-dependent signaling, neoplastic activities, and cross-talk with EGFR and HER-3. Clin Cancer Res.

[16] Moser C, Ruemmele P, Gehmert S, Schenk H, Kreutz MP, Mycielska ME (2012). STAT5b as molecular target in pancreatic cancer–inhibition of tumor growth, angiogenesis, and metastases. Neoplasia.

[17] Taeger J, Moser C, Hellerbrand C, Mycielska ME, Glockzin G, Schlitt HJ (2011). Targeting FGFR/PDGFR/VEGFR impairs tumor growth, angiogenesis, and metastasis by effects on tumor cells, endothelial cells, and pericytes in pancreatic cancer. Mol Cancer Ther.

[18] Lang SA, Schachtschneider P, Moser C, Mori A, Hackl C, Gaumann A (2008). Dual targeting of Raf and VEGF receptor 2 reduces growth and metastasis of pancreatic cancer through direct effects on tumor cells, endothelial cells, and pericytes. Mol Cancer Ther.

[19] Lang SA, Moser C, Gaumann A, Klein D, Glockzin G, Popp FC (2007). Targeting heat shock protein 90 in pancreatic cancer impairs insulin-like growth factor-I receptor signaling, disrupts an interleukin-6/signal-transducer and activator of transcription 3/hypoxia-inducible factor-1alpha autocrine loop, and reduces orthotopic tumor growth. Clin Cancer Res.

[20] Lang SA, Moser C, Fichnter-Feigl S, Schachtschneider P, Hellerbrand C, Schmitz V (2009). Targeting heat-shock protein 90 improves efficacy of rapamycin in a model of hepatocellular carcinoma in mice. Hepatology.

[21] Giroux V, Malicet C, Barthet M, Gironella M, Archange C, Dagorn JC (2006). p8 is a new target of gemcitabine in pancreatic cancer cells. Clin Cancer Res.

[22] Michl P, Gress TM (2013). Current concepts and novel targets in advanced pancreatic cancer. Gut.

[23] Lu Z, Kleeff J, Shrikhande S, Zimmermann T, Korc M, Friess H (2000). Expression of the multidrug-resistance 1 (MDR1) gene and prognosis in human pancreatic cancer. Pancreas.

[24] Zhou J, Liu M, Aneja R, Chandra R, Lage H, Joshi HC (2006). Reversal of P-glycoprotein-mediated multidrug resistance in cancer cells by the c-Jun NH2-terminal kinase. Cancer Res.

[25] Neesse A, Michl P, Frese KK, Feig C, Cook N, Jacobetz MA (2011). Stromal biology and therapy in pancreatic cancer. Gut.

[26] Di Renzo MF, Poulsom R, Olivero M, Comoglio PM, Lemoine NR (1995). Expression of the Met/hepatocyte growth factor receptor in human pancreatic cancer. Cancer Res.

[27] Paciucci R, Vila MR, Adell T, Diaz VM, Tora M, Nakamura T (1998). Activation of the urokinase plasminogen activator/urokinase plasminogen activator receptor system and redistribution of E-cadherin are associated with hepatocyte growth factor-induced motility of pancreas tumor cells overexpressing Met. Am J Pathol.

[28] Avan A, Caretti V, Funel N, Galvani E, Maftouh M, Honeywell RJ (2013). Crizotinib inhibits metabolic inactivation of gemcitabine in c-Met-driven pancreatic carcinoma. Cancer Res.

[29] Christensen JG, Schreck R, Burrows J, Kuruganti P, Chan E, Le P (2003). A selective small molecule inhibitor of c-Met kinase inhibits c-Met-dependent phenotypes in vitro and exhibits cytoreductive antitumor activity in vivo. Cancer Res.

[30] Jin H, Yang R, Zheng Z, Romero M, Ross J, Bou-Reslan H (2008). MetMAb, the one-armed 5D5 anti-c-Met antibody, inhibits orthotopic pancreatic tumor growth and improves survival. Cancer Res.

[31] Ucar DA, Magis AT, He DH, Lawrence NJ, Sebti SM, Kurenova E (2013). Inhibiting the interaction of cMET and IGF-1R with FAK effectively reduces growth of pancreatic cancer cells in vitro and in vivo. Anticancer Agents Med Chem.

[32] Ide T, Kitajima Y, Miyoshi A, Ohtsuka T, Mitsuno M, Ohtaka K (2007). The hypoxic environment in tumor-stromal cells accelerates pancreatic cancer progression via the activation of paracrine hepatocyte growth factor/c-Met signaling. Ann Surg Oncol.

[33] Ying JE, Zhu LM, Liu BX (2012). Developments in metastatic pancreatic cancer: is gemcitabine still the standard?. World J Gastroenterol.

[34] Shah AN, Summy JM, Zhang J, Park SI, Parikh NU, Gallick GE (2007). Development and characterization of gemcitabine-resistant pancreatic tumor cells. Ann Surg Oncol.

[35] Li C, Wu JJ, Hynes M, Dosch J, Sarkar B, Welling TH (2011). c-Met is a marker of pancreatic cancer stem cells and therapeutic target. Gastroenterology.

[36] Bentires-Alj M, Barbu V, Fillet M, Chariot A, Relic B, Jacobs N (2003). NF-kappaB transcription factor induces drug resistance through MDR1 expression in cancer cells. Oncogene.

[37] Zhang W, Chen H, Liu DL, Li H, Luo J, Zhang JH (2013). Emodin sensitizes the gemcitabine-resistant cell line Bxpc-3/Gem to gemcitabine via downregulation of NF-kappaB and its regulated targets. Int J Oncol.

[38] Doublier S, Belisario DC, Polimeni M, Annaratone L, Riganti C, Allia E (2012). HIF-1 activation induces doxorubicin resistance in MCF7 3-D spheroids via P-glycoprotein expression: a potential model of the chemo-resistance of invasive micropapillary carcinoma of the breast. BMC Cancer.

[39] Matsumura A, Kubota T, Taiyoh H, Fujiwara H, Okamoto K, Ichikawa D (2013). HGF regulates VEGF expression via the c-Met receptor downstream pathways, PI3K/Akt, MAPK and STAT3, in CT26 murine cells. Int J Oncol.

[40] Ding Y, Cravero JD, Adrian K, Grippo P (2010). Modeling pancreatic cancer in vivo: from xenograft and carcinogen-induced systems to genetically engineered mice. Pancreas.

