# Supplementary material for: Targeting cMET with INC280 impairs tumour growth and improves efficacy of gemcitabine in a pancreatic cancer model
Source: BMC Cancer. 2015 Feb 19;15:71. doi: 10.1186/s12885-015-1064-9 (PMC4340491; doi:10.1186/s12885-015-1064-9)
Supplement: Additional file 1: Figure S1. — Effects of targeting cMET on L3.6pl pancreatic cancer cells. A) Incubation of L3.6pl cells with the cMET inhibitor INC280 has no effect on constitutive growth. When cells were stimulated with HGF, a significant improvement of growth was observed (#P<0.05). This was abrogated by cMET inhibition with INC280 (*P<0.05). B) HGF induces cancer cell motility (#P<0.05) that can efficiently be blocked by INC280 (*P<0.05). Constitutive motility remains unaffected. C) Treatment with INC280 disrupts HGF-mediated phosphorylation of Akt and ERK after 4 and 24 hours of treatment. Bars=SEM. [file 12885_2015_1064_MOESM1_ESM.pptx]

## Slide 1
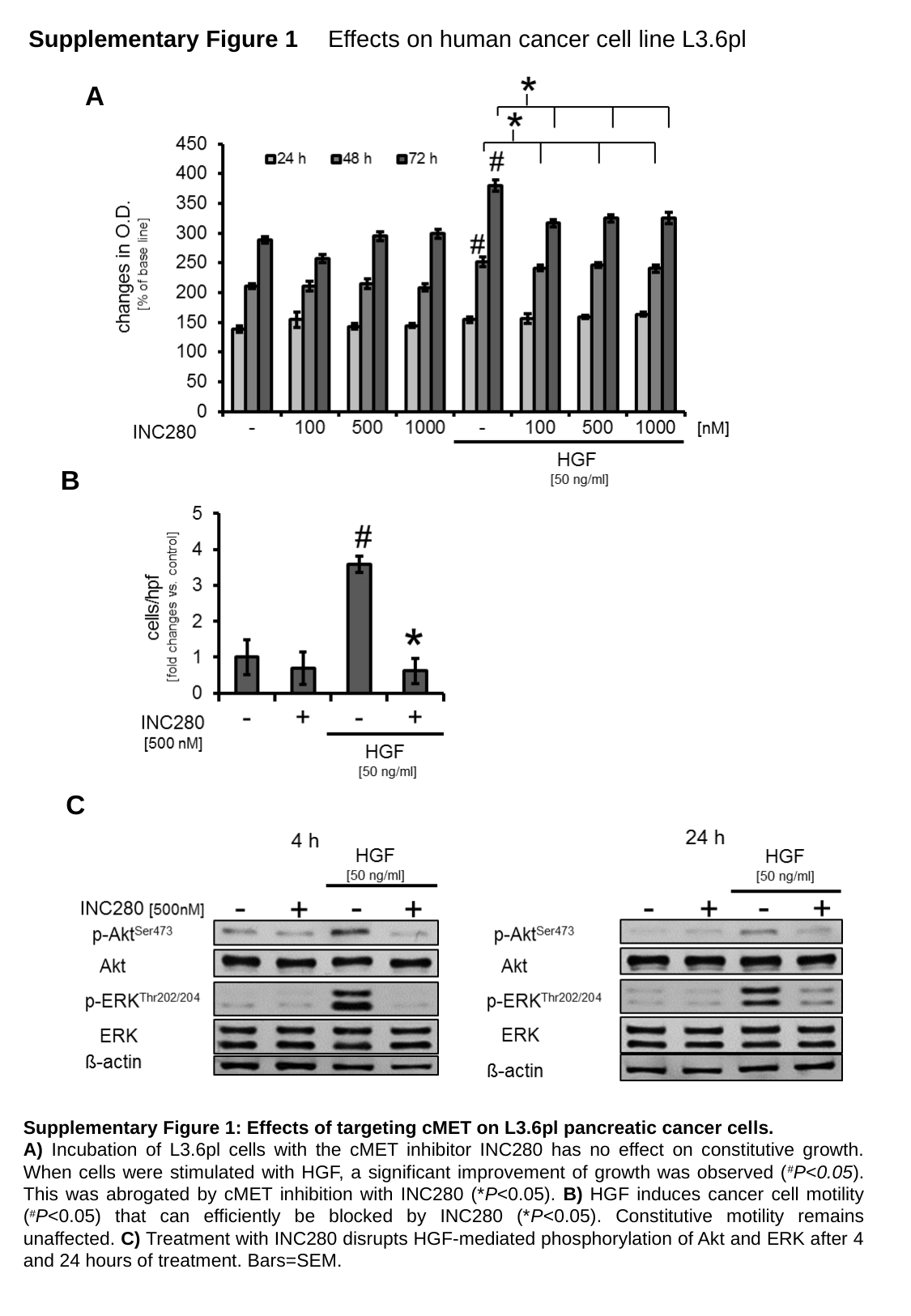

Supplementary Figure 1
Effects on human cancer cell line L3.6pl
A
B
C
Supplementary Figure 1: Effects of targeting cMET on L3.6pl pancreatic cancer cells.
A) Incubation of L3.6pl cells with the cMET inhibitor INC280 has no effect on constitutive growth. When cells were stimulated with HGF, a significant improvement of growth was observed (#P<0.05). This was abrogated by cMET inhibition with INC280 (*P<0.05). B) HGF induces cancer cell motility (#P<0.05) that can efficiently be blocked by INC280 (*P<0.05). Constitutive motility remains unaffected. C) Treatment with INC280 disrupts HGF-mediated phosphorylation of Akt and ERK after 4 and 24 hours of treatment. Bars=SEM.
