# Supplementary material for: Targeting cMET with INC280 impairs tumour growth and improves efficacy of gemcitabine in a pancreatic cancer model
Source: BMC Cancer. 2015 Feb 19;15:71. doi: 10.1186/s12885-015-1064-9 (PMC4340491; doi:10.1186/s12885-015-1064-9)
Supplement: Additional file 2: Figure S2. — Effects of targeting cMET on VEGF-A and PDGF-B secretion from cancer cells. A) Hypoxia led to a significant increase in VEGF-A secretion from L3.6pl cancer cells (#P<0.05). INC280 did not affect this. B) Induction with DFX led to a significant increase in PDGF-B secretion from HPAF-II pancreatic cancer cells (#P<0.05). Targeting cMET had no impact on this. C) Similar, DFX led to increase in PDGF-B secretion from L3.6pl and INC280 did not reduce this in vitro (#P<0.05). D) Gemcitabine resistant MiaPaCa2(G250) showed a higher secretion of PDGF-B compared to regular MiaPaCa2(par) (#P<0.05). cMET inhibition had no effect on this in vitro. E) Incubation with DFX led to a significant increase in PDGF-B secretion from MiaPaCa2(G250) but INC280 did not affect this increase (#P<0.05). Bars=SE. [file 12885_2015_1064_MOESM2_ESM.pptx]

## Slide 1
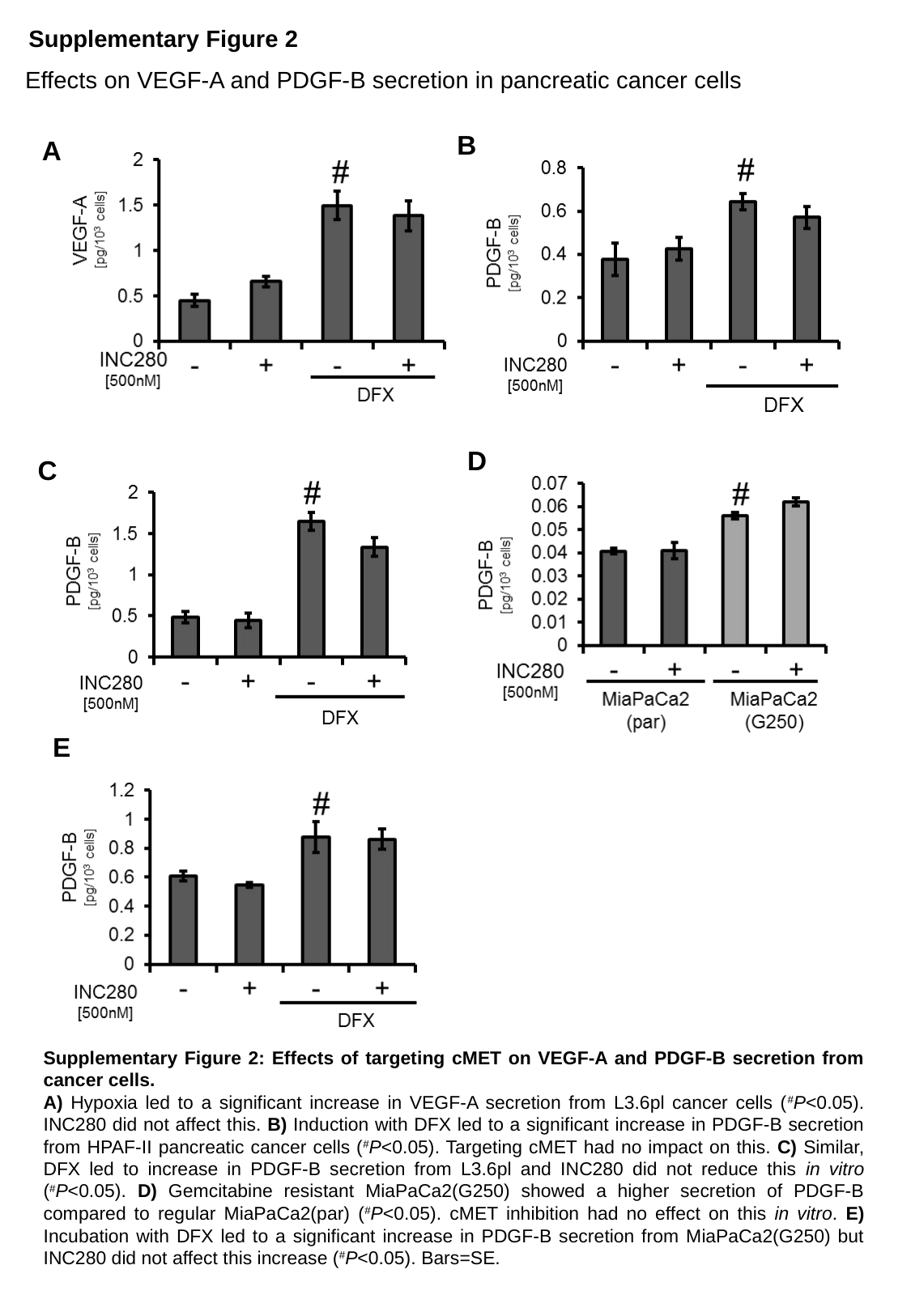

Supplementary Figure 2
Effects on VEGF-A and PDGF-B secretion in pancreatic cancer cells
B
A
D
C
E
Supplementary Figure 2: Effects of targeting cMET on VEGF-A and PDGF-B secretion from cancer cells.
A) Hypoxia led to a significant increase in VEGF-A secretion from L3.6pl cancer cells (#P<0.05). INC280 did not affect this. B) Induction with DFX led to a significant increase in PDGF-B secretion from HPAF-II pancreatic cancer cells (#P<0.05). Targeting cMET had no impact on this. C) Similar, DFX led to increase in PDGF-B secretion from L3.6pl and INC280 did not reduce this in vitro (#P<0.05). D) Gemcitabine resistant MiaPaCa2(G250) showed a higher secretion of PDGF-B compared to regular MiaPaCa2(par) (#P<0.05). cMET inhibition had no effect on this in vitro. E) Incubation with DFX led to a significant increase in PDGF-B secretion from MiaPaCa2(G250) but INC280 did not affect this increase (#P<0.05). Bars=SE.
