# Supplementary material for: Targeting cMET with INC280 impairs tumour growth and improves efficacy of gemcitabine in a pancreatic cancer model
Source: BMC Cancer. 2015 Feb 19;15:71. doi: 10.1186/s12885-015-1064-9 (PMC4340491; doi:10.1186/s12885-015-1064-9)
Supplement: Additional file 3: Figure S3. — Effects of targeting cMET on ECs and VSMCs. A) No effect of targeting cMET with INC280 on ECs was detected upon constitutive conditions. B) HGF induces growth of ECs after 48 and 72 hours of incubation (#P<0.05). Incubation with INC280 impairs this (*P<0.05). C) In VSMCs, INC280 led to inhibition of constitutive growth upon constitutive conditions (*P<0.05). D) Upon serum-starved conditions, no effect on constitutive growth was detectable. Similar, stimulation of VSMCs with HGF did not affect in vitro growth. Bars = SE. [file 12885_2015_1064_MOESM3_ESM.pptx]

## Slide 1
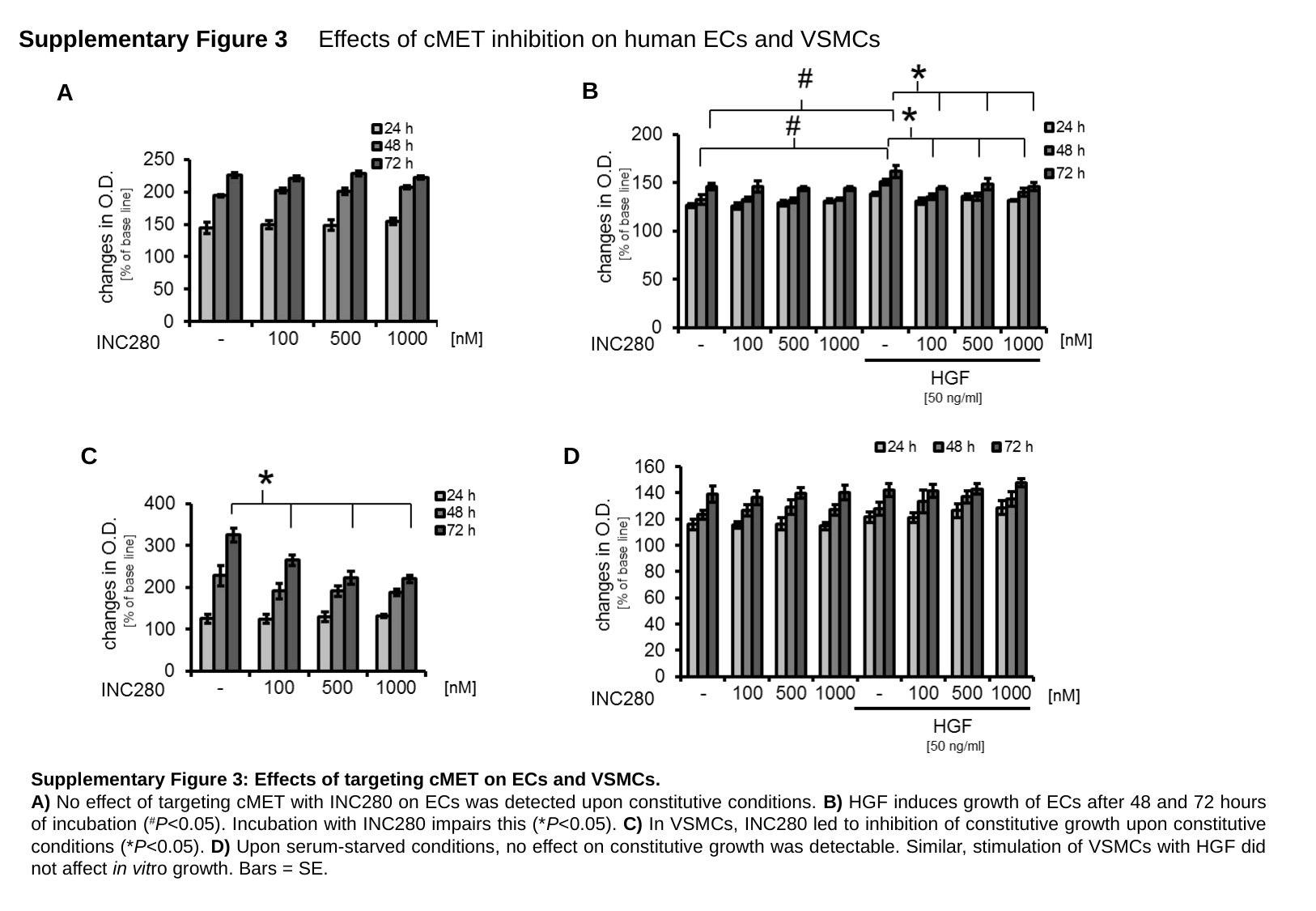

Supplementary Figure 3
Effects of cMET inhibition on human ECs and VSMCs
B
A
C
D
Supplementary Figure 3: Effects of targeting cMET on ECs and VSMCs.
A) No effect of targeting cMET with INC280 on ECs was detected upon constitutive conditions. B) HGF induces growth of ECs after 48 and 72 hours of incubation (#P<0.05). Incubation with INC280 impairs this (*P<0.05). C) In VSMCs, INC280 led to inhibition of constitutive growth upon constitutive conditions (*P<0.05). D) Upon serum-starved conditions, no effect on constitutive growth was detectable. Similar, stimulation of VSMCs with HGF did not affect in vitro growth. Bars = SE.
